# 14.85 µW Analog Front-End for Photoplethysmography Acquisition with 142-dBΩ Gain and 64.2-pA_rms_ Noise

**DOI:** 10.3390/s19030512

**Published:** 2019-01-26

**Authors:** Binghui Lin, Mohamed Atef, Guoxing Wang

**Affiliations:** 1 Department of Microelectronics, Shanghai Jiao Tong University, Shanghai 200240, China; linbinghuihenry@sjtu.edu.cn; 2 MoE Key Lab of Artificial Intelligence, AI Institute, Shanghai Jiao Tong University, Shanghai 200240, China; 3 Electrical Engineering Department, Assiut University, Assiut 71516, Egypt; moh_atef@aun.edu.eg

**Keywords:** analog front-end, low power, photoplethysmography, transimpedance amplifier

## Abstract

A low-power, high-gain, and low-noise analog front-end (AFE) for wearable photoplethysmography (PPG) acquisition systems is designed and fabricated in a 0.35 μm CMOS process. A high transimpedance gain of 142 dBΩ and a low input-referred noise of only 64.2 pA_rms_ was achieved. A Sub-Hz filter was integrated using a pseudo resistor, resulting in a small silicon area. To mitigate the saturation problem caused by background light (BGL), a BGL cancellation loop and a new simple automatic gain control block are used to enhance the dynamic range and improve the linearity of the AFE. The measurement results show that a DC photocurrent component up-to-10 μA can be rejected and the PPG output swing can reach 1.42 V_pp_ at THD < 1%. The chip consumes a total power of 14.85 μW using a single 3.3-V power supply. In this work, the small area and efficiently integrated blocks were used to implement the PPG AFE and the silicon area is minimized to 0.8 mm × 0.8 mm.

## 1. Introduction

**Figure 1:**
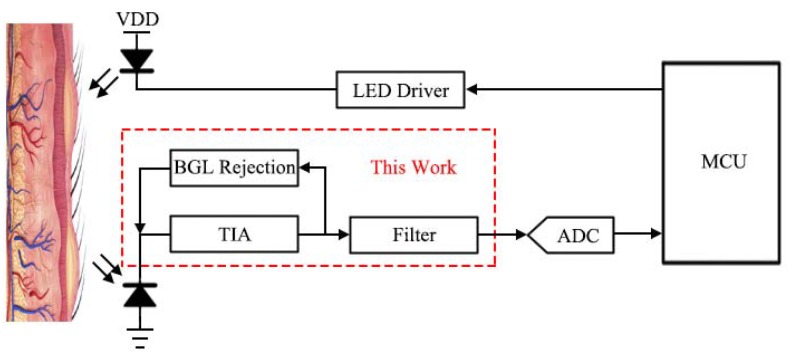
The system diagram of a typical PPG monitoring application.

The Photoplethysmography (PPG) signal can be used for many applications such as oxyhemoglobin saturation (SpO_2_) measurement, diagnosis of cardiovascular diseases, heart rate and blood pressure (BP) [1,2,3,4,5]. The PPG signal is usually acquired using optical transceivers. The transmitter consists of a (few) LED(s) and associated driving circuits and the receiver usually consists of a (few) photodiode(s) (PD) followed by the transimpedance amplifier (TIA) and other signal processing circuits [3]. The light from the LEDs is shone onto the skin and reflected back by (or transmitted through) the flesh. The reflected/transmitted light signal contains the information of the blood flow volume change and is picked up by a (few) PD(s). As shown in Figure 1, the photocurrent of the PPG signal is usually converted (amplified) to a voltage and fed to an analog-to-digital converter (ADC) and then processed in the digital domain by the Microcontroller Unit (MCU) [6].

The proposed system is used for BP application and only the AC component of the PPG signal is needed for blood pressure calculation [6]. When reducing the current of LEDs, the AC component of the PPG signal can be down to 1 nA. To maintain the strong amplitude and SNR of the output PPG signal, the transimpedance gain should be larger than 140 dBΩ and the input-referred noise should be less than 100 pA_rms_. In real complex measurement conditions, the DC component of the input photocurrent can be up to 10 µA so the AFE should be designed to reject such a large photocurrent.

The AFE circuit is very important because it determines the quality of the sensed PPG signal, which affects the accuracy of the monitoring system. There are several problems when designing the PPG AFE. Firstly, the power consumption of the AFE should be as low as possible, especially if it is used in wearable devices where the battery life is critical. To reduce the power of the whole system, it has been proposed to operate the LEDs in a duty-cycled mode, i.e., to turn on the LEDs only for a small portion of the time, such as 1% [7]. In such systems, the power consumption of LEDs can be reduced a lot and thus is comparable to that of the AFE or even lower [7]. In our proposed work, to reduce the power consumption, subthreshold design techniques are used and the simple circuit architecture has been explored. Even though the circuits work in a continuous mode, most circuits can be reused in a duty-cycled operation with slight modifications. Secondly, the noise of the acquisition circuit should be low to be able to capture weak signals. In another way, strong light can be used to obtain a strong signal but at the cost of high power (25 to 150 mA) [8]. In this design, two amplifying stages were implemented to achieve high gain and low input-referred noise, thus the power consumption of the LED can be reduced. Thirdly, the PPG signal is usually from 0.5 Hz to 4 Hz [9], so a band-pass filter with low-frequency corners should be designed to eliminate out-of-band noise, which requires large resistances and/or capacitances. Some designs used off-chip capacitors while others [10] realized the large resistance on-chip with switched-capacitor circuits, requiring additional circuits and increasing the power consumption. In our proposed work, pseudo resistors with extremely large on-chip resistance values were used to implement the filters and the silicon area cost is minimized. The fourth issue of PPG acquisition is the background light (BGL) effect since the desired AC signal induced by the blood volume changes is usually 0.5% to 1.5% of the DC component (undesired) induced by the BGL [11]. The large DC input photocurrent saturates the TIA easily and needs to be canceled. For canceling the DC photocurrent component, References [12,13] used a single BGL cancellation loop to reject the DC current automatically and Reference [14] used a dual loop to reject BGL. However, References [10,15] tackled this problem by using a DAC to generate the rejecting current to mitigate the BGL effect. It requires complex digital circuits to control a DAC so it consumes more power. In our proposed work, a single analog BGL cancellation loop, together with simple automatic gain control (AGC), has been designed and the dynamic range is extended.

This paper introduces an integrated PPG sensor with low power, high sensitivity and wide dynamic range. An AGC is integrated to increase the sensing dynamic range. A DC photocurrent cancellation loop is proposed to prevent the receiver saturation at high PPG signal values.

In this work, the circuit design to address the above challenges will be discussed in detail in Section 2. The chip has been fabricated and the measurement results will be given in Section 3 and, finally, the conclusion will be given in Section 4.

## 2. Circuit Design

### 2.1. System Architecture

**Figure 2:**
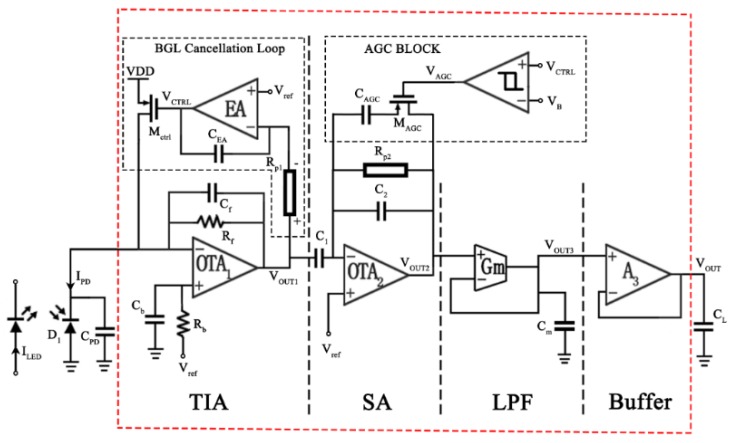
The proposed architecture of the AFE.

The proposed AFE has four stages, as shown in Figure 2. The first stage (TIA) converts the weak photocurrent from PD ($I_{PD}$) into a voltage $V_{OUT1}$ with a high transimpedance gain (122 dBΩ). The BGL cancellation loop is implemented in this stage to reject the input DC photocurrent. The second stage, named the secondary amplifier (SA), is implemented with capacitive feedback using OTA_2_ with a closed-loop gain of 20 dB. The third stage is a low-pass filter (LPF) to reject the noise at a high frequency. The fourth stage is a unity-gain buffer to drive the off-chip load for testing.

In order to reduce the power consumption, most circuits are designed to work in the subthreshold region. In addition, the AFE should be high-gain and low-noise to detect weak photocurrents. This can relax the required light strength so the total power of the LEDs can be reduced. To achieve low input-referred noise, a large transimpedance gain of TIA is adopted. However, the large transimpedance gain of the first stage TIA may easily lead to saturation if the large DC photocurrent due to BGL is not taken care of. Therefore, a BGL cancellation loop is designed to reject the DC photocurrent. In addition, an AGC scheme is designed in the second stage. When the photocurrent is very large, the AGC block can lower the gain of SA by half, preventing the distortion of the PPG signal. With the BGL cancellation loop and AGC block, the dynamic range of the AFE is enhanced. As will be discussed in Section 2.1 and Section 2.2, the resistance of $R_{p1}$ and $R_{p2}$ should be very large, at least a few hundred gigaohms, to realize the extremely large time constants for filters with low-frequency corners. To minimize the number of off-chip components, pseudo resistor structures are realized on-chip with a small silicon area.

### 2.2. The Proposed TIA Topology

The TIA, as shown in Figure 2, is the most critical block where both low noise and high gain are required to improve the quality of the acquired signal. Ignoring the BGL cancellation loop first, the transfer function of the TIA can be derived as Equation (1).

(1) $$Z_{TIA0}\left(s\right)=-R_{f}\frac{1}{1+sC_{f}R_{f}}$$

where $R_{f}$ and $C_{f}$ are the feedback resistor and capacitor, respectively.

**Figure 3:**
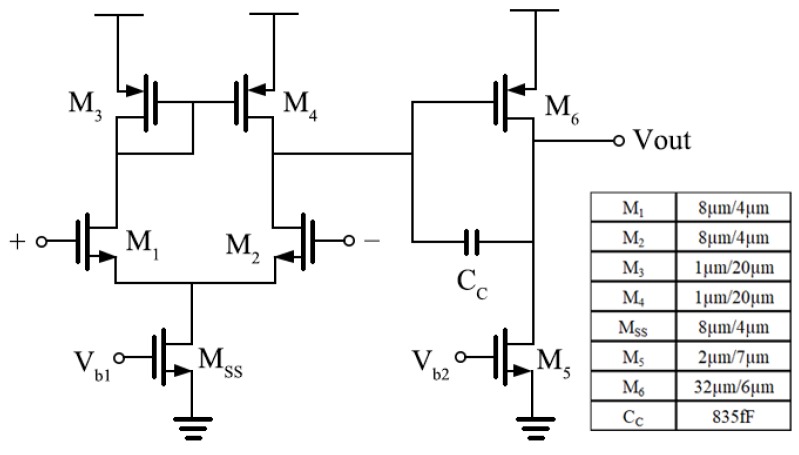
The schematic of OTA_1_.

The input-referred noise $\bar{I_{n,in}^{2}}$ can be calculated from Equation (2) assuming $R_{f}$ is large enough so that the noise from the following stages can be ignored.

(2) $$\bar{I_{n,in}^{2}}\approx 8kT\gamma \left(\frac{1}{g_{m1,2}}+\frac{g_{m3,4}}{g_{m1,2}^{2}}\right)+\frac{4kT}{R_{f}}$$

where $g_{m1,2}$ and $g_{m3,4}$ are the transconductances of M_1,2_ and M_3,4_ (in Figure 3) respectively, *k* is the Boltzmann constant, *T* is the absolute temperature in Kelvin, and γ is the effective noise coefficient which is around 0.67 for long-channel MOSFETs.

**Figure 4:**
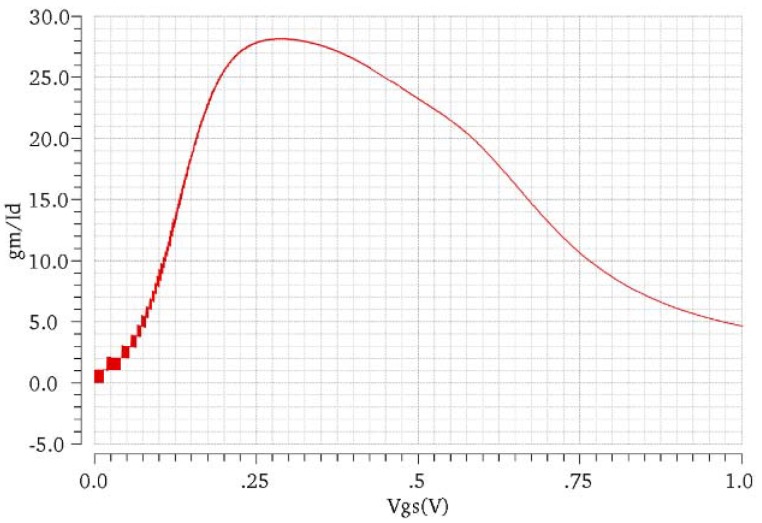
$\frac{g_{m}}{I_{d}}$ at different $V_{gs}$ of NMOS in the 0.35 µm process (W/L = 1 µm/1 µm).

The circuit topology of OTA_1_ is shown in Figure 3. A traditional two-stage operational amplifier is used and the sizes of the transistors are also shown in Figure 3. In OTA_1_, M_1_ and M_2_ are designed to work in the subthreshold region, which means the gate to source voltage, $V_{gs}$, is very close to the threshold voltage, $V_{th}$. In this condition, the MOSFET will develop a weak inversion layer and a higher $\frac{g_{m}}{I_{d}}$ can be achieved. From Figure 4, the highest $\frac{g_{m}}{I_{d}}$ is achieved when $V_{gs}$ is around 300 mV but the overdrive voltage is too small since $V_{th}$ is nearly 600 mV for NMOS in the 0.35 µm process. For M_1,2_, the $V_{gs}$ is designed as about 500 mV to get a high transconductance with a low bias current maintaining that the MOSFET is always working in the subthreshold region. From the simulation results, the DC gain is 121 dB (>>1), the unity-gain frequency is 300 kHz and the input-referred noise (integrated from 0 to 10 Hz) is 29 µVrms. The total bias current is 2.45 µA.

From Equation (2), it can be observed that an efficient way to achieve low noise and high gain is to use a large $R_{f}$. However, if $R_{f}$ is too large, OTA_1_ may be easily saturated because the input photocurrent contains a large DC component which is also amplified. To mitigate the impact of BGL, the DC rejection circuit should be applied. References [10,15] sense the output voltage and use digital circuits and DACs to generate the DC current to reject the input DC current. In this way, complex digital circuits should be designed, which may consume more power. As a result, an analog BGL cancellation loop is added to TIA to suppress the DC component of the photocurrent, as shown in Figure 2. In the loop, the error amplifier (EA) senses the output voltage of OTA_1_ and controls the drain current of M_ctrl_ to compensate for the input DC photocurrent of TIA. When a large photocurrent is induced by a strong BGL, $V_{OUT1}$ will become higher. The $V_{CTRL}$ will decrease and thus the $V_{sg}$ of M_ctrl_ will increase, resulting in a higher DC current passing through M_ctrl_. As a result, the photocurrent from the PD will be compensated so that only the AC component pass through $R_{f}$. Thus, the output voltage of OTA_1_ is maintained around $V_{ref}$, preventing the circuit from saturation. $C_{b}$ and $R_{b}$ are used to balance the input impedance of OTA_1_.

This BGL cancellation loop is negative voltage-current feedback. This feedback loop is for DC or very-low-frequency (<0.5 Hz) photocurrents because only the photocurrent from BGL should be rejected. $C_{EA}$ and $R_{p1}$ are used to connect the EA as an integrator which functions as a low-pass filter. To realize the filter, an extremely large time constant should be implemented to generate a pole between DC and 0.5 Hz (the PPG signal is from 0.5 to 4 Hz). The transfer function of the first stage with the BGL cancellation loop can be derived as Equation (3) and the high-pass pole due to the loop can be expressed as Equation (4), assuming the gain of EA, $A_{v,EA}$, is much larger than 1.

(3) $$Z_{TIA}\left(s\right)\approx -R_{f}\cdot \frac{1+sC_{EA}R_{p1}A_{v,EA}}{1+sC_{EA}R_{p1}A_{v,EA}+R_{f}g_{m,ctrl}A_{v,EA}}\cdot \frac{1}{1+sC_{f}R_{f}}$$

(4) $$\omega_{p}\left(s\right)=\frac{1+R_{f}g_{m,ctrl}A_{v,EA}}{C_{EA}R_{p1}A_{v,EA}}$$

where $g_{m,ctrl}$ is the transconductance of M_ctrl_, $A_{v,EA}$ is the open-loop gain of EA.

**Figure 5:**
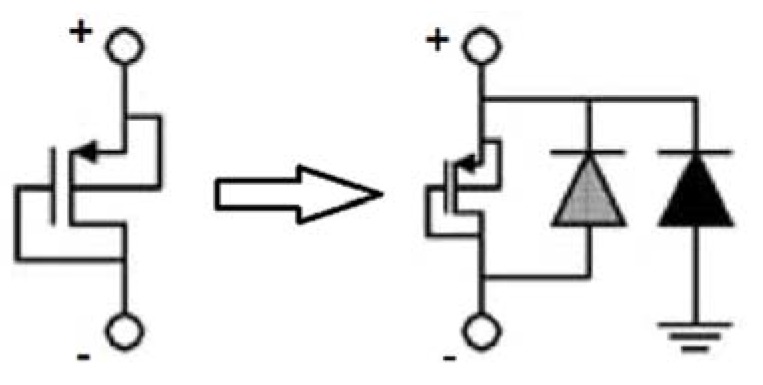
The pseudo resistor used for $R_{p1}$.

From Equation (4), $C_{EA}$ and $R_{p1}$ should be large enough to push the pole to a low frequency (<0.5 Hz). We choose an on-chip $C_{EA}$ of 65 pF, so $R_{p1}$ should be hundreds of gigaohms. To implement such a large resistance on-chip, pseudo resistors should be used [16] since the switch capacitor scheme in Reference [10] needs additional digital control. Figure 5 shows the structure of the pseudo resistor adopted for $R_{p1}$. When a positive voltage is applied to $R_{p1}$, $V_{sg}$ of the MOSFET is a little larger than zero and the MOSFET works in the weak inversion region, which determines the equivalent resistance. When a negative voltage is applied instead, the equivalent resistance is mainly determined by the weak forward-biased diodes. When the two terminals’ voltages are close to each other, which is determined by the feedback loop, the pseudo resistor can provide the desired resistance.

**Figure 6:**
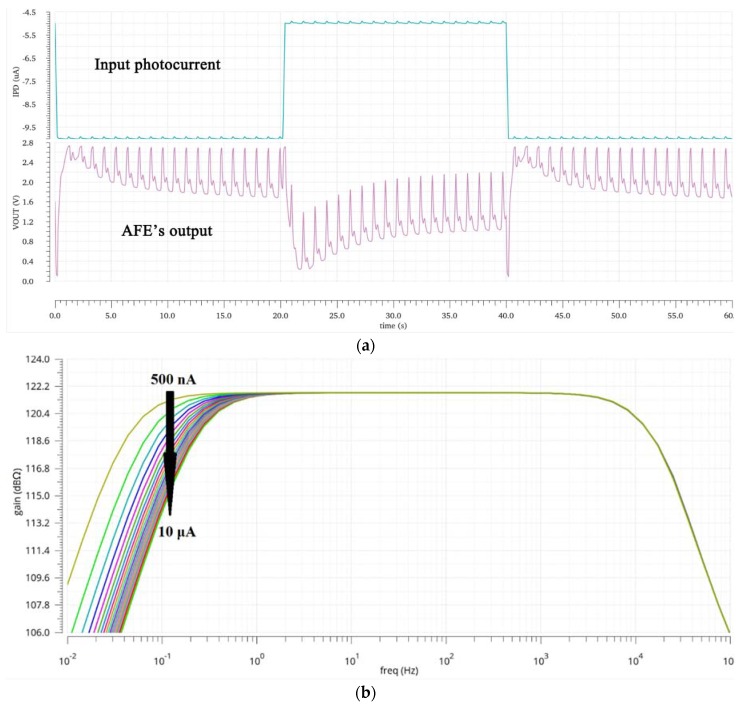
The simulation result of TIA. Transient response (**a**) and frequency response (**b**) with different BGL levels. In (a), ${I_{PD}}^{\prime }s$ DC level changes between 5 and 10 μA while the PPG signal is maintained as 0.1 μA peak-to-peak.

To verify the function of the BGL cancellation loop, different $I_{PD}$ values are applied and the simulation results are shown in Figure 6. From Figure 6a, it can be seen that with the BGL cancellation loop, the PPG signals are amplified even though its amplitude is much less than the BGL changes. Without BGL cancellation, the PPG signals will get saturated due to the strong BGL. The simulated frequency response is shown as Figure 6b. With the BGL cancellation loop, the transimpedance gain of TIA at the passband of the desired PPG signal remains relatively constant around 122 dBΩ even for an $I_{PD}$ up to 10 μA. As the $I_{PD}$ increases, the high-pass corner frequency also increases as can be explained by Equation (4) since $g_{m,ctrl}$ increases as the compensation current increases.

### 2.3. Secondary Amplifier (SA)

The second stage is a capacitive feedback amplifier, with its transfer function of $A_{v2}\left(s\right)$ shown in Equation (5). $R_{p2}$ offers the DC bias voltage to the negative input of OTA_2_. The SA’s high-pass corner frequency should be less than 0.5 Hz to further reject the BGL. This requires the pole, determined by $\frac{1}{C_{2}R_{p2}}$ to be less than 0.5 Hz.

(5) $$A_{v2}\left(s\right)=-\frac{sC_{1}R_{p2}}{1+sC_{2}R_{p2}}$$

**Figure 7:**
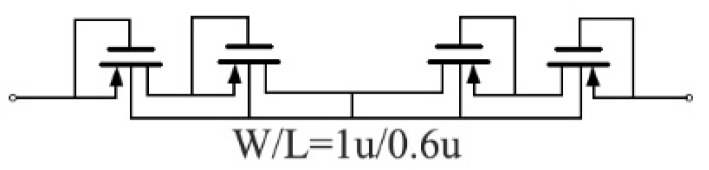
The pseudo resistor used for $R_{p2}$.

A pseudo resistor, shown in Figure 7, is adapted to realize $R_{p2}$ with a resistance of about 1.7 TΩ. Considering the large variation of the pseudo resistor, the pole is designed to be 15 MHz, much less than 0.5 Hz for a safety margin. In this design, $C_{1}$ is 63 pF and $C_{2}$ is 6.3 pF, achieving another 20-dB voltage gain. Since a high output swing is also needed in OTA_2_, it is designed using the same architecture as OTA_1_ but biased at a lower current of 300 nA.

As the photocurrent from PD, including the DC and AC component, varies with lighting, skin and sensing positions, the signal strength may change a lot. A simple AGC block is designed to automatically adjust the gain of SA. When the input signal is too strong, the BGL cancellation loop decreases the voltage of $V_{CTRL}$. Once $V_{CTRL}$ is lower than the preset voltage of $V_{B}$, the comparator output $V_{AGC}$ is low and turns on the transistor M_AGC_. As a result, $C_{2}$ is paralleled with $C_{AGC}$, which increases the total feedback capacitance and decreases the SA’s gain. $C_{AGC}$ is the same as $C_{2}$, and therefore, the gain can be reduced by half for large inputs, preventing the AC signal from saturation.

**Figure 8:**
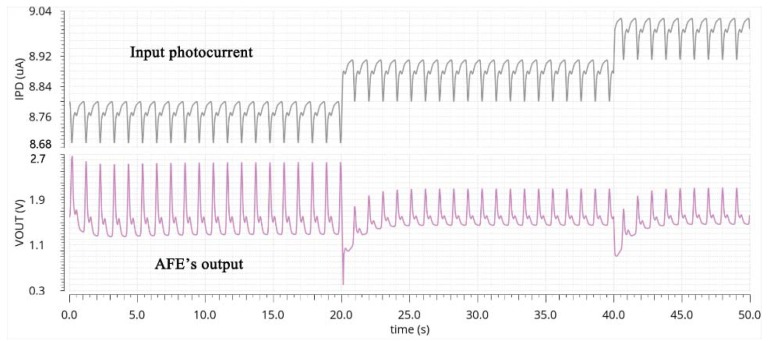
The transient response (simulation) of the final output as the DC component of $I_{PD}$ increases.

Figure 8 shows the functioning of the AGC. The DC level of $V_{OUT}$ varies little as the DC component of $I_{PD}$ increases. When the DC level reaches a high level of 8.8 μA, the gain of the AFE is decreased by 6 dB and the amplitude of the final output is also decreased by half, preventing the signal from saturation.

### 2.4. Gm-C Filter

**Figure 9:**
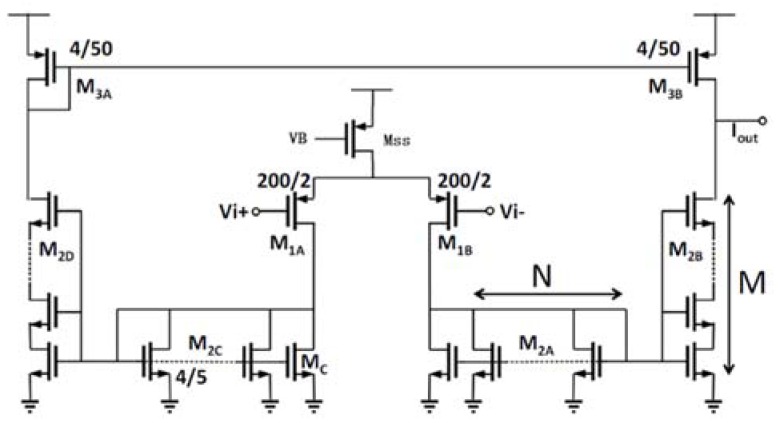
The schematic of the Gm block [17].

The third stage, a low-pass filter, filters out the out-of-band noise and the 3-dB frequency is chosen as 25 Hz. A Gm-C architecture is adopted, as shown in Figure 2. In order to realize a small equivalent transconductance of the Gm block, the series-parallel (SP) architecture shown in Figure 9 [17] is adopted. *M* and *N* are both chosen as 20 so that the equivalent transconductance is reduced by 400 (*M* × *N*) times that of M_1A,B_. With the 72-nA biasing current of Mss, the equivalent transconductance of the Gm block is 3 nS. As a result, $C_{m}$ can be reduced to 18.63 pF which is implemented using an on-chip capacitor.

## 3. Measurement Results

**Figure 10:**
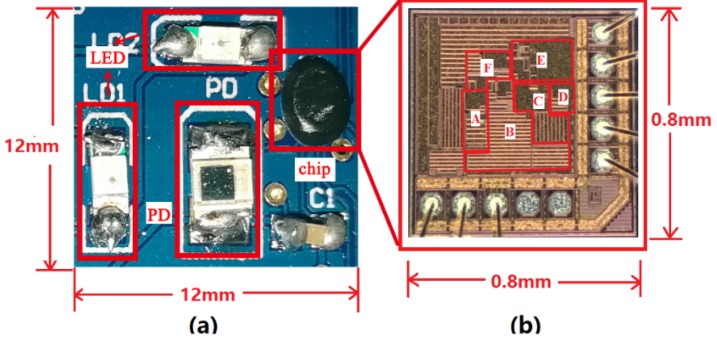
The testing board (**a**) and micrograph of the die (**b**), including TIA (A), SA (B), LPF (C), buffer (D), biasing (E) and AGC (F).

The proposed design is fabricated in a 0.35 μm standard CMOS process. The testing board is shown in Figure 10a and the die photograph is shown in Figure 10b. The chip is directly bonded onto the PCB. The used LEDs are red, and the model is 15-21/R6C-AN1P2/2T. The model of the PD is PD15-22C/TR8. 

**Figure 11:**
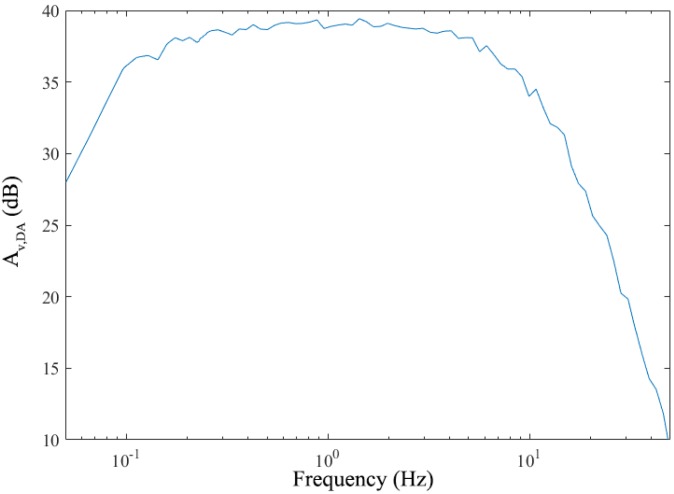
The measured frequency response of $A_{v,DA}$.

The AC component of the photocurrent is hard to be measured and thus the frequency response of the AFE is difficult to be measured directly. A dynamic analyzer (Keysight 35670A, Agilent, Santa Clara, CA, USA) is used to generate a voltage of $V_{source}$. The current from the LEDs, $I_{LED}$, is controlled by $V_{source}$ through a discrete circuit. To mitigate the influence from the ambient light, the testing board is put in a black box. The output signal of AFE, $V_{out}\left(f\right)$, is recorded and the voltage gain, $A_{v,DA}\left(f\right)$, defined as Equation (6), is drawn in Figure 11, whose shape is close to the frequency response of the AFE, assuming $I_{LED}$ to be linear to $V_{source}$ and $I_{PD}$ to be linear to guire $I_{LED}$. It shows that the passband is from 0.1 Hz to 10 Hz which covers the frequency range of the PPG signal as desired. 

**Figure 12:**
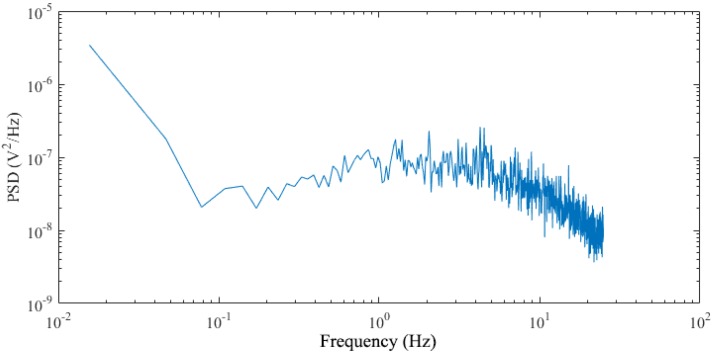
The noise response of the AFE.

Putting the testing board in a black box, the input photocurrent of the AFE is nearly zero and the Fourier transform of the output is shown in Figure 12. Integrating the output noise voltage power from 0.1 Hz to 10 Hz (the passband of the AFE), the output noise is 809.4 μV_rms_ and the input-referred noise is 64.2 pA_rms_.

(6) $$A_{v,\mathrm{DA}}(f)=\frac{V_{out}\left(f\right)}{V_{source}\left(f\right)}$$

**Figure 13:**
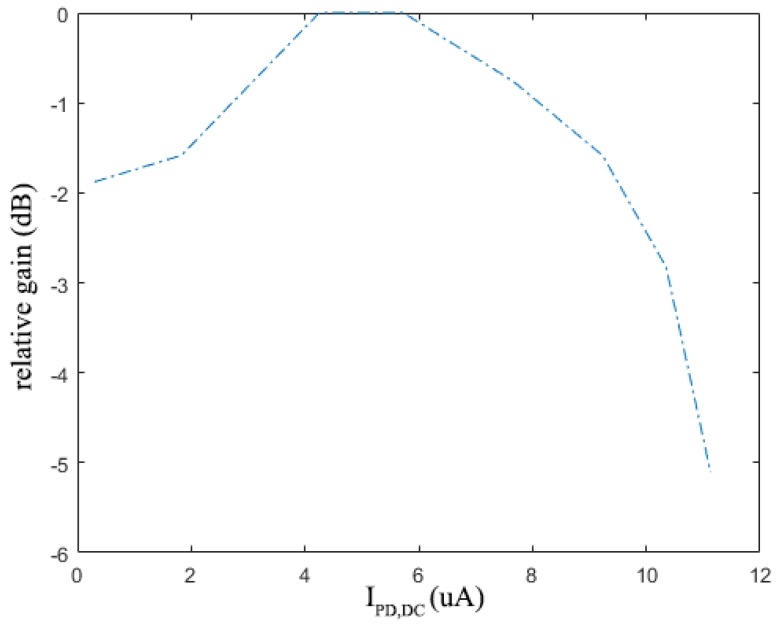
The transimpedance gain (in dB), relative to the nominal value of 142 dBΩ, at different DC input photocurrent values.

The relative transimpedance gain (the ratio of the actual transimpedance gain to the nominal value of 142 dBΩ) at 1 Hz with different input DC photocurrents are measured and shown in Figure 13. $I_{PD,DC}$ is the DC value of input photocurrent which is measured by the digital multimeter, Keysight 34461A. When the input photocurrent is larger than 10 μA, the gain is decreased by 3 dB, which shows that the BGL cancellation loop works well and is able to reject the input DC photocurrent up to 10 μA.

The power supply voltage can go down when the battery gets weak. In order to measure the working range of the chip, a 1-Hz sine wave with a 0.15-µA offset and a small amplitude is applied to the AFE. When the power supply is less than 2 V, it takes more than 10 s to settle down. When the power supply is less than 1.7 V, the output distorts heavily. The process limits the maximum supply voltage to 3.7 V so that the working range of the AFE is from 2 V to 3.7 V. At the nominal supply voltage of 3.3 V, the measured power consumption is 14.85 μW.

**Figure 14:**
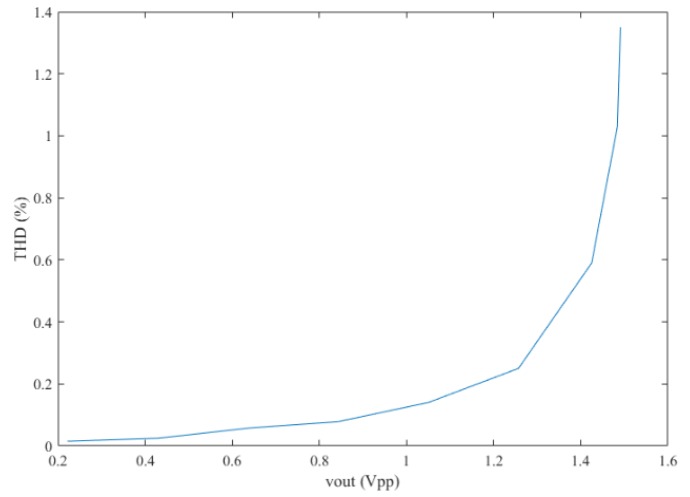
The THD of the AFE with different peak-to-peak outputs (when the input photocurrent is 1.12 µA and the signal is a 1-Hz sine wave with a small amplitude).

Figure 14 shows the THD at different peak-to-peak output voltages with the sine wave’s input. It shows that the THD is less than 1% when the output signal is less than 1.42 V_pp_ with the typical photocurrent. When the AGC is off and the 5.2-µA photocurrent is applied to the AFE, the peak-to-peak output voltage is 1.49 V and the THD is 0.65%. While turning on the AGC, the peak-to-peak output voltage is 0.79 V and the THD is 0.012%, which shows that the AGC can decrease the gain and improve the linearity.

**Figure 15:**
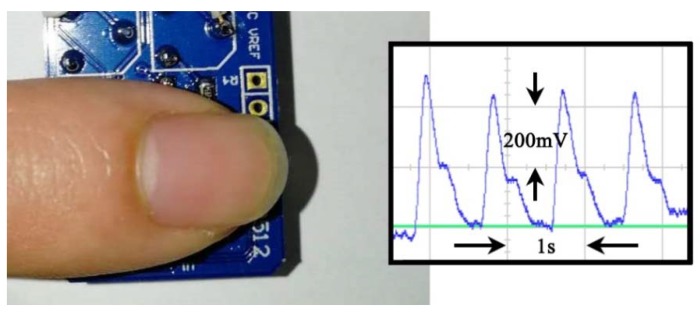
The output PPG signal measured on the finger.

**Figure 16:**
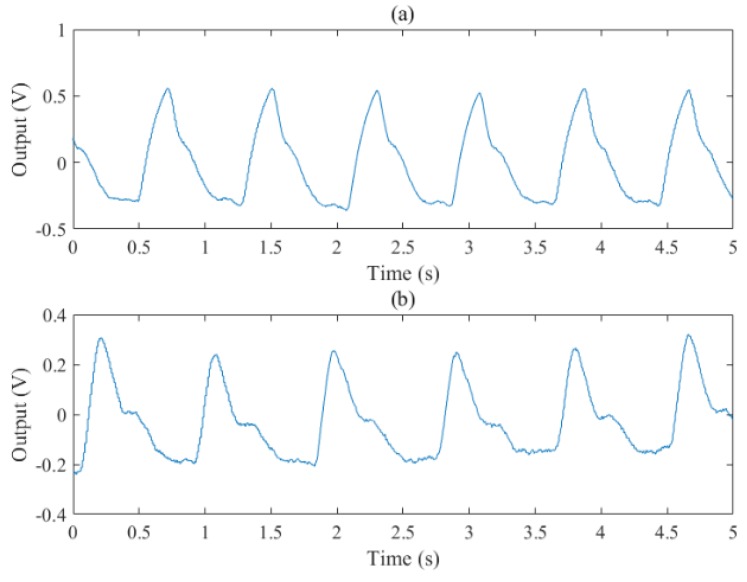
The output PPG signal with a large input photocurrent when the AGC is off (**a**) and on (**b**).

The chip has been tested to acquire finger PPG signals, as shown in Figure 15 and Figure 16. A finger is put on the sensor module and the reflected light can be detected using an oscilloscope as shown on the right of Figure 15. It can be seen that the PPG waveform is clear and the two peaks and valley (normally important features) are evident. To verify the function of the AGC block, a strong LED light is applied and the measured waveforms are shown in Figure 16. With the AGC on, the gain is decreased by 6 dB for large inputs and the feature points are reserved better than that with the AGC off.

Table 1 shows the comparison of this work with recently published works. FoM_1_ shows the trade-off between power consumption and noise performance while FoM_2_ shows the trade-off between transimpedance gain and DC rejection current. The weaker signal can be detected if the transimpedance gain becomes larger but the DC photocurrent can also be amplified and leads the circuit to saturation. Since the DC rejection current shows the maximum input photocurrent, transimpedance gain and DC rejection current should be traded off. The design in Reference [7] has a gain of 145 dBΩ and a DC current rejection up to 70 μA at the cost of a high power consumption of 3.36 mW. Reference [15] demonstrated the lowest input-referred noise of 15.4 pA_rms_, but its gain of 127 dBΩ is lower and its power consumption of 135 μW is higher than this work. Compared to other designs published recently and the commercial product AFE4403, the proposed AFE in this work has the lowest power consumption of 14.85 μW and the smallest area of 0.64 mm^2^ (even the oldest technology of 0.35 μm is adopted). Besides, the transimpedance gain and input-referred noise show advantages while the weak point of this design is that it can only work in the continuous mode.

## 4. Discussions and Conclusions

An integrated low-power AFE with a high gain and low noise for PPG sensing is presented. In the proposed design, the analog voltage-current feedback loop with a pseudo resistor is adopted and is able to reject up-to-10 µA of DC photocurrent with low power consumption. In addition, a simple AGC block can adjust the gain of SA according to the input photocurrent. Both the BGL cancellation loop and the AGC block can prevent the circuits from saturation and enhance the DR of the AFE in low power budget cost. From the measurement results, the high-gain TIA and the on-chip filter reduce the input-referred noise to only 64.2 pA_rms_, which allows the AFE to detect weaker signals and lowers the power consumption of the optical transmitter. The measurement results have shown that the power consumption of the proposed AFE is 14.85 μW so the life of the battery can be extended widely. The proposed AFE works in the continuous mode, which can be further improved with the duty cycled operation. From the system level aspect, the power consumption is dominated by the LEDs which may consume dozens of milliwatts. If the sensor works in the pulsative operation, the power consumption of the LEDs can be much reduced. A new chip with the pulsative operation has been designed and is being fabricated. Additionally, the current prototype uses a 12-bit ADC embedded in the MCU (CY8C4247LQI-BL483) with a maximum sampling frequency of 1 MHz (exceeding the need of the PPG acquisition in the proposed application). The proposed AFE has a small area but a fully customized chip integrated with ADC, digital control and wireless transmission will further lower the power and reduce the form factor of the device.

## Figures and Tables

**Table 1:** The comparison with recently published work.

Design	This Work	[7]	[8]	[10]	[15]	[18]	[19]
Process (μm)	0.35	0.35	0.065	0.18	0.18	0.13	N.A.
Supply Voltage (V)	2.0–3.7	2.7–4	1.2	1.2	1.2/1.8	1.2	2.0–3.6
Operation Mode	Continuous	Pulse	Pulse	Pulse	Pulse	Continuous	Pulse
Gain $R_{T}$ (dBΩ)	142/135	145	52	N.A.	84–127	130.9	80–132
Bandwidth (Hz)	10	50	20k	3.5	64	70	500
Input-Referred Noise $\sqrt{\bar{I_{n,in}^{2}}}$ (pA_rms_)	64.2	79	N.A.	486	15.4	260	5.3
Power Consumption $P_{T}$ (μW)	14.85	3360	340^1^	172^2^	135	26.4	676.5
DC Current Rejection $I_{R}$ (μA)	0–10	0–70	N.A.	0–10	0–10	0–30	0–10
Area (mm^2^)	0.64	1.36^3^	2.08^3^	5^3^	1.57^3^	3.84^3^	N.A.
FoM_1_^4^ (1/(μW·nA_rms_^2^))	16.36	0.048	N.A.	0.025	31.17	0.58	52.83
FoM_2_^5^ (dBV)	42	62	N.A.	N.A.	27	41	32

^1^ Includes AFE and ADC; ^2^ Includes AFE, ADC and digital block; ^3^ Estimated from the die photo from the paper; ^4^ FoM_1_ is calculated as $\frac{1}{P_{T}\;\times \;\bar{I_{n,in}^{2}}}$; ^5^ FoM_2_ is calculated as $R_{T}\;\times \;I_{R}$.
